# Proposal for probing energy transfer pathway by single-molecule pump-dump experiment

**DOI:** 10.1038/srep27535

**Published:** 2016-06-09

**Authors:** Ming-Jie Tao, Qing Ai, Fu-Guo Deng, Yuan-Chung Cheng

**Affiliations:** 1 Department of Physics, Applied Optics Beijing Area Major Laboratory, Beijing Normal University, Beijing 100875, China; 2 Department of Chemistry, Center for Quantum Science and Engineering, National Taiwan University, Taipei City 106, Taiwan

## Abstract

The structure of Fenna-Matthews-Olson (FMO) light-harvesting complex had long been recognized as containing seven bacteriochlorophyll (BChl) molecules. Recently, an additional BChl molecule was discovered in the crystal structure of the FMO complex, which may serve as a link between baseplate and the remaining seven molecules. Here, we investigate excitation energy transfer (EET) process by simulating single-molecule pump-dump experiment in the eight-molecules complex. We adopt the coherent modified Redfield theory and non-Markovian quantum jump method to simulate EET dynamics. This scheme provides a practical approach of detecting the realistic EET pathway in BChl complexes with currently available experimental technology. And it may assist optimizing design of artificial light-harvesting devices.

As the chemical energy that all life on earth demand is almost from solar energy harvested by virtue of photosynthesis, many researchers devote themselves into improving the production of photosynthesis. In recent decades, much attention has been focused on excitation energy transfer (EET) in photosynthesis, that is, photosynthetic complexes transmit efficiently the solar energy captured in the peripheral light-harvesting antenna to reaction centres. Although the pathways and time scales of EET are often described by semiclassical models^1^,^2^, we still lack precise mechanism responsible for efficient EET. Recently, quantum coherence effects in photosynthetic EET were predicted^3^,^4^ and indirectly observed^5^. In particular, as revealed by two experiments in 2007^6^,^7^, the quantum coherence of EET in natural photosynthesis has attracted more and more interest from broad fields, such as physical society, chemical society, and biological society. The quantum coherence manifests itself in the wavelike evolution of exciton states^6^,^8^. In photosynthetic systems, the quantum coherence between electronic excitations plays an important role in the optimization of EET efficiency^9^. Although much progress has been made in revealing quantum coherence effects in the photosynthetic EET, there is an important issue under heated debate: although it seems that there exists the quantum coherent oscillation in site populations of Fenna-Matthews-Olson (FMO)^10^,^11^,^12^, others challenged this discovery as it depended on the EET pathway and number of bacteriochlorophylls (BChls) in FMO^8^,^13^,^14^,^15^.

The FMO pigment-protein complex, found in low light-adapted green sulfur bacteria^8^,^16^, has become an important model system to study EET in photosynthesis^5^,^6^,^8^,^9^,^13^,^14^,^15^,^16^,^17^,^18^. Savikhin *et al*. observed hint for quantum beating in the FMO complex by means of pump-probe anisotropy techniques^5^. Engel *et al*. studied the FMO complex isolated from *Chlorobium tepidum* with 2D electronic spectroscopy and gained direct evidence of long-lived electronic coherence^6^. Recently, the effects of quantum coherence on enhancement of photosynthetic EET efficiency were discussed from the perspective of quantum walk by Aspuru-Guzik and coworkers, and by Plenio and Huelga^19^,^20^,^21^,^22^, respectively. As we know, the FMO complex is a trimer made of identical subunits, and it had long been recognized that there are seven BChl molecules in each monomeric subunit. However, the presence of an additional BChl pigment in each subunit was reported recently, which had probably been lost in the previous recrystallization. In 2011, Busch *et al*. suggested the eighth pigment being the linker between the baseplate and the remaining seven BChls by crystallographic studies and calculations of the optical properties of the FMO^13^. In the same year, Ritschel *et al*. theoretically investigated EET in the full FMO trimer, and focused on the role of BChl 8 in the energy transfer on different sets of transition energies. It was shown that BChl 8 plays an important role in receiving excitation from the outer light harvesting antenna^14^. Meanwhile, Moix *et al*. studied the influence of the 8th BChl on the dynamics in FMO through the generalized Bloch-Redfield equation and the noninteracting blip approximation^15^. And it was discovered that the EET in eight-BChls complex remains efficient and robust, although the existence of the 8th BChl clearly affects the energy transfer pathways as revealed by the simulated 2D electronic spectroscopy^23^. Nevertheless, these previous studies did not come to an agreement on whether the energy flow in FMO complex passes through site 8 or not. On the other hand, various approaches have been put forward to optimize the energy transfer in the eight-BChls FMO, e.g., by introducing phases in inter-site couplings^24^, and by tuning temperature of the baseplate^25^. At this stage, two questions naturally come to our mind, when we consider designing artificial light-harvesting^26^: whether is it necessary to include an additional BChl in artificial light-harvesting device if the efficiency is not essentially affected by its presence? How can we determine whether a specific BChl is in an EET pathway or not?

Among the methods capable of detecting the ultrafast quantum dynamics in EET, the 2D electronic spectroscopy is a four-wave-mixing photon-echo approach to provide valuable information about electronic transitions^6^,^16^. However, as its signal is averaged over an ensemble of inhomogeneous photosynthetic complexes, it may not be a competent candidate for revealing the EET pathway. A recently-developed single molecule technique has grown into a powerful method for exploring the individual nanoscale behavior of molecules in complex local environments^27^,^28^. In this sense, the single-molecule pump-dump experiment provides useful insights into the ultrafast quantum dynamics of the photosynthetic complex^29^,^30^, and thus can be used to settle this issue of EET pathway whether site 8 is in the energy transfer pathway of FMO. In 2005, Barbara *et al*. investigated the molecular structure and charge-transfer dynamics of conjugated polymers through the single-molecule spectroscopy^31^. In 2009, Gerhardt *et al*. observed 5 cycles of Rabi oscillations in a single molecule via narrow zero-photon transition using short laser pulses^32^. During the past decade, van Hulst and coworkers developed single-molecule pump-probe techniques to control vibrational wave packets and coherent energy transfer over different pathways in individual molecules at ambient conditions^29^,^33^,^34^, although it might not be easy to distinguish single molecule coherence oscillations from spectral interference^35^. These technical advances^36^,^37^,^38^ inspire us to propose detecting the EET pathway in FMO by the single-molecule pump-dump experiment.

Traditionally, the EET is described in two opposite regimes, i.e. Förster and Redfield theories respectively^39^. In order to simulate the single-molecule experiment in natural photosynthetic complexes, we explore a theoretical approach^40^ by combining a coherent modified Redfield theory (CMRT)^41^,^42^ and a non-Markovian quantum jump (NMQJ) method^43^,^44^,^45^. As a generalization of the modified Redfield theory^46^,^47^, the CMRT can describe a quantum system’s density matrix completely^40^ and has been successfully applied to simulate coherent EET dynamics in photosynthetic light harvesting^41^,^42^,^48^. In 2008, Piilo and coworkers developed an efficient NMQJ method to simulate non-Markovian dynamics of an open quantum system^44^,^45^. These developments inspire us to efficiently unravel a set of equations of motion for density matrix by the NMQJ method^49^,^50^,^51^. In order to utilize the NMQJ method, we rewrite the master equation of CMRT in the Lindblad form^40^. With the help of this approach, we theoretically simulate the quantum dynamics in the single-molecule experiment and obtain some useful results to determine whether or not the EET in FMO passes through site 8.

## Results

### EET-path-resolved experiment

**Figure 1:**
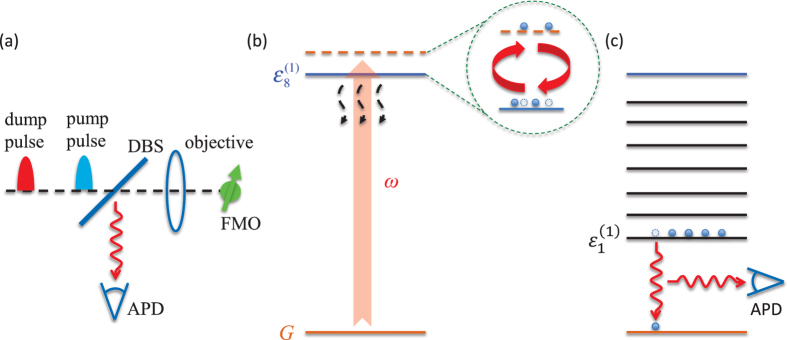
(**a**) Schematic diagram of single-molecule pump-dump experimental setup. (**b**) Schematic energy diagram when the dump pulse is applied: there will be induced transition between the highest-exciton state 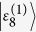 and up-lifted ground state |*G*〉 in the rotating frame. Since the induced-couplings between the ground state and lower-exciton states are relatively smaller with respect to their level spacings, they will result in small shifts to the effective energies. Therefore, it is equivalent to a dissipative two-level system. (**c**) After the pulse ends, the population of the exciton states will relax to the lowest exciton state from which they jump to ground state with a photon emitted.

As illustrated in Fig. 1(a), we present a proposal for settling the problem whether a site lies within the EET pathway in a natural photosynthetic complex by single-molecule pump-dump experiment. A mode-locked laser or optical parametric oscillator offers the visible laser pulse to induce coherent transitions between the ground state and exciton states of the FMO complex. The pulse train enters the confocal microscopy which contains a dichroic beam-splitter (DBS) and an objective and an avalanche photodiode (APD). Having passed a DBS, the pulse is focused on a single FMO complex by an oil immersion objective. The emitted fluorescence is collected by the same objective. After reflected by the DBS, the fluorescence is detected by an APD. For detailed information about the experiment setup, please refer to ref. 33.

The single-molecule pump-dump (probe) technique developed by van Hulst and coworkers has been successfully applied to resolving the ultrafast EET dynamics at physiological conditions^36^,^37^. It can effectively control vibrational wave packets and demonstrate interference among different EET pathways in a single photosynthetic complex^29^,^34^. Most importantly, a femtosecond single-qubit operation can be carried out on single molecules at room temperature^38^, which implies potential application of quantum information^52^,^53^ on photosynthetic light-harvesting. In this regard, the single-molecule pump-dump technique is chosen to resolve the EET pathway in a photosynthetic light-harvesting.

#### Assumptions and initial-state preparation

Our experimental scheme is based on the following assumptions: Without loss of generality, the initial excitation of FMO is prepared at site 1 or site 8 for the sake of simplicity in our numerical simulation. In order to realize this assumption, the total system is made up of an FMO, a baseplate, and an antenna. The initial excitation is prepared at the antenna by absorbing a photon at the frequency with a blue shift to the highest exciton state of FMO. Furthermore, the antenna is spatially far away from the FMO in order not to excite the FMO during the preparation process when the pump pulse is applied. Since the total Hamiltonian including the FMO and the baseplate and outer antenna is unknown, the initial excitation at site 1 or site 8 in FMO is utilized to simplify numerical simulation. Moreover, we further assume that the site energy for the eighth site of FMO is the highest of all sites. This seems to be true according to simulations made in ref. 13. Due to its small electronic couplings to other sites, the highest exciton state is a localized eigen state of FMO Hamiltonian. In this case, the energy flow through site 1 only will not essentially pass through site 8.

#### Experimental scheme

As shown in Fig. 1(a), based on the above assumptions, we propose the following experimental scheme to determine the EET path in FMO, including the main procedures as follows:

After photoexcitation by the pump pulse, a single-molecule FMO evolves freely from the initial site, i.e., site 1 or site 8. Then, a dump pulse begins to be applied to the FMO molecule at time *t*_1_. Its driving frequency *ω* is chosen to be in close resonance with the transition from the ground state to the highest exciton state, meanwhile it is largely detuned from the transitions to other exciton states. In this case, the population on the target state can be coherently transferred to the ground state, while the population on the other exciton states can be nearly undisturbed. The dump pulse ends at time *t*_2_ and then the FMO molecule is left to evolve freely again. Due to the coupling to the phonon bath, the remaining population on the single-excitation subspace quickly relaxes to the lowest exciton state without emitting a photon. Finally, the population on the lowest exciton state transits to the ground state with fluorescence detected by photon detector. Generally speaking, the relaxation within the single-excitation subspace, e.g. 0.1 ~ 10 ps, is much faster than dissipation to the ground state, e.g. ~1 ns. Therefore, we would expect that all the population on the higher exciton states could reach the lowest exciton state before they emit a fluorescent photon.

#### Parameter optimization

Since we aim at transferring the population on the highest exciton state 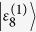 to the ground state 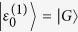, we should tune the driving frequency *ω* in close resonance with the target state 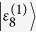 while let it be largely detuned from other exciton states 
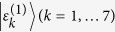
. In order to fulfill this requirement, the driving frequency is chosen as the one with a blue shift to the transition between 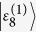 and 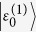, i.e., 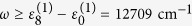.

Furthermore, the electric polarization of the dump pulse is chosen at the direction perpendicular to both 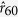 and 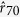 where 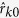 is the unit vector of the transition dipole between the *k*th exciton state and the ground state^15^, i.e., 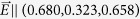. In this case, there will induce the couplings between the ground state and the exciton states with strengths


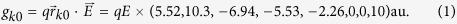


The transition dipole orientations 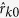 can be measured by the reduced linear dichroism signal, which is calculated from two orthogonal polarizations detected in the in-plane of the sample^54^. This technique was theoretically proposed by Fourkas^54^, and experimentally realized by Vacha^55^. On account of the large-detuning condition, i.e., 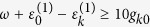 for *k* = 1,…7, the maximum coupling between the highest exciton state and the ground state is *g*_80_ = 35 cm^−1^. Notice that a strong Rabi frequency as large as 318 cm^−1^ was realized in experiments^33^. Since the typical transition dipoles of BChls are of the order of several Debyes, the maximum coupling induced by laser fields is achievable in practice. For numerical simulations, the information for all molecular electric dipoles 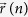 are provided in the Appendix.

### Theoretical simulation method

We adopt a Frenkel exciton model to describe photoexcitations in the FMO complex^16^. The model includes electronic interactions between any two sites of FMO, and the system Hamiltonian is^15^


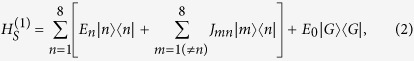


where |*n*〉 is the state with single-excitation on the *n*th site, *E*_*n*_ is the corresponding site energy, and *J*_*mn*_ is the electronic coupling between site *m* and *n*. Besides, there is no excitation on the ground state |*G*〉 with energy *E*_0_ and there is no direct coupling between it and the single-excitation states. We adopt the effective Hamiltonian for the 8-sites FMO proposed by Moix *et al*., which provides excellent description of the spectra and EET dynamics of the complex. The explicit form of Hamiltonian 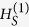 is given in the Appendix.

To describe the EET dynamics induced by the system-bath couplings, we could obtain the master equation for the CMRT as^41^,^42^


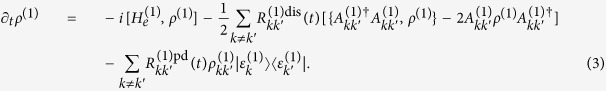


Here 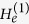 governs the coherent evolution of the EET. It owns the same eigen states 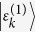 as 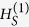 but with different eigen energies 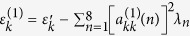, where the eigen energies 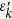 of 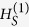 are modified by the reorganization energies induced by the system-bath couplings, and 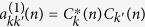 is the overlap of *k*th and *k*′th eigen states at site *n*. Notably, the equation of motion is in a generalized Lindblad form^40^ with the jump operators defined as 
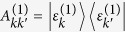
. Based on the CMRT, the dissipation and pure-dephasing rates are respectively


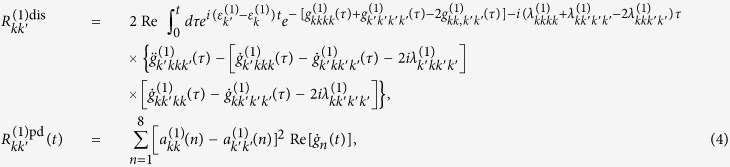


where


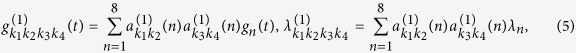



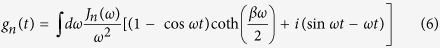


is the lineshape function. *λ*_*n*_ and *J*_*n*_(*ω*) are the reorganization energy and spectral density of the *n*th molecule, respectively. *β* = 1/*k*_*B*_*T* with *k*_*B*_ and *T* are Boltzman constant and temperature, respectively. In our numerical simulations, we assume identical reorganization energy *λ* = 35 cm^−1^ and identical Ohmic spectral density *J*(*ω*) = *λ*(*ω*/*ω*_*c*_)exp(−*ω*/*ω*_*c*_) with cut-off *ω*_*c*_ = 50 cm^−1^ for all molecules and the experiment is conducted at ambient temperature, i.e. *T* = 300 K.

In order to implement the NMQJ method, we rewrite Eq. (3) in the Lindblad form as^40^


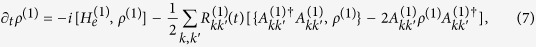


where the matrix element of the rates is defined as


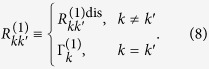


The dephasing rates are given by


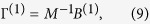


where the matrix elements of *B*^(1)^ and *M* are respectively


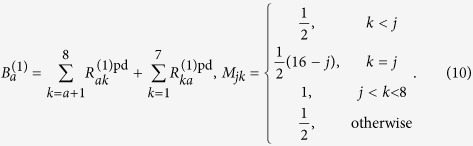


As shown in Fig. 1(b), after the free evolution, there is a dump pulse with frequency *ω* applied to the FMO molecule and thus transitions between the ground state 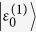 and delocalized exciton states 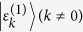 are induced. In this situation, the electronic Hamiltonian reads


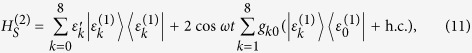


where 2*g*_*k*0_ is the laser-induced coupling strength between the ground state 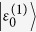 and the delocalized exciton state 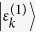.

Transformed to a rotating frame with 
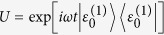
, the effective Hamiltonian of the electronic part 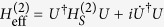 is


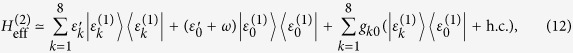


where we have dropped the fast-oscillating terms with factors exp(±*i*2*ωt*). The above Hamiltonian can be diagonalized as 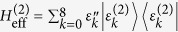, where 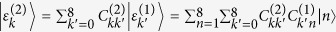 is the eigen state with eigen energy 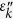.

In the basis of 
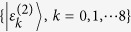
 of the rotating frame, we could obtain the master equation of the same form as Eq. (3), but the system Hamiltonian is replaced by 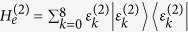 with eigen energies 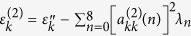. The dissipation and pure-dephasing rates can be calculated in the same way as Eq. (4) but 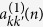 is substituted by 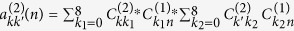. As a consequence, for the duration with a pulse, the master equation can also be rewritten in the Lindblad form and thus be solved by the NMQJ approach. It is worthy of mentioning that the calculated density matrix should be transformed back to the static frame as 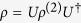. Note that in order to test experimentally the EET pathway through site 8 in the FMO, here we assume the laser frequency is in resonance with 
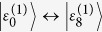
. However, our formulism is general and could be applied to other resonance conditions.

### Results and analysis

In the previous section, we briefly introduced a newly-developed CMRT-NMQJ approach^40^,^41^ to simulate the quantum dynamics of an FMO complex in a single-molecule pump-dump experiment, as presented in Fig. 1.

#### Exact result

Since a portion of population of the exciton states has been transferred to the ground state during the dump pulse duration, the detected fluorescence intensity is determined by the population on the ground state, which can be controlled by the following parameters, i.e., the Rabi frequency *g* ≡ *g*_80_, the beginning time of the laser pulse *t*_1_, the pulse width *T* = *t*_2_ − *t*_1_, and the frequency of drive *ω*. Before the numerical simulation, we define the fluorescence quantum yield as


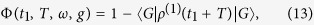


which is the total population on the single-excitation subspace right after the dump pulse ends. As the laser field is tuned in close resonance with the selected energy level, the fluorescence quantum yield is very sensitive to the irradiation condition. In order to clearly illustrate this phenomenon and also compare the effects of different initial states, for a given range of experimental parameters, we can define the visibility of fluorescence quantum yield as


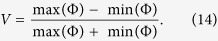


**Figure 2:**
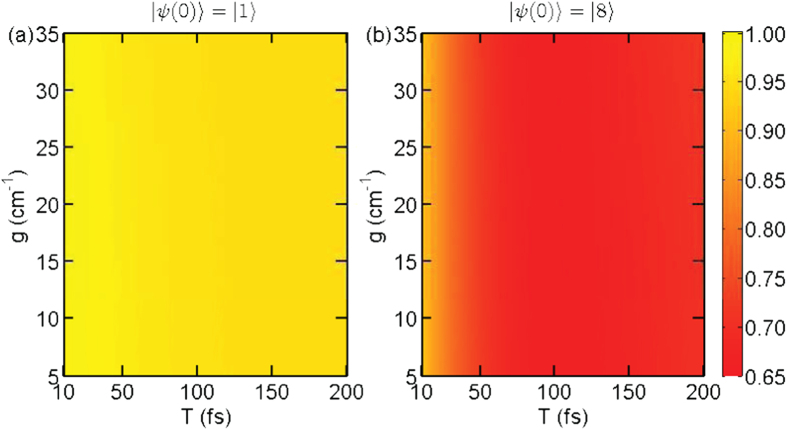
Quantum yield Φ vs the pulse duration *T* and Rabi frequency *g* for the driving frequency *ω* = 12709 cm^−1^ and the beginning time *t*_1_ = 50 fs: (**a**) initial state |*ψ*(0)〉 = |1〉 with observed visibility *V* = 0.02; (**b**) |*ψ*(0)〉 = |8〉 with *V* = 0.16.

**Figure 3:**
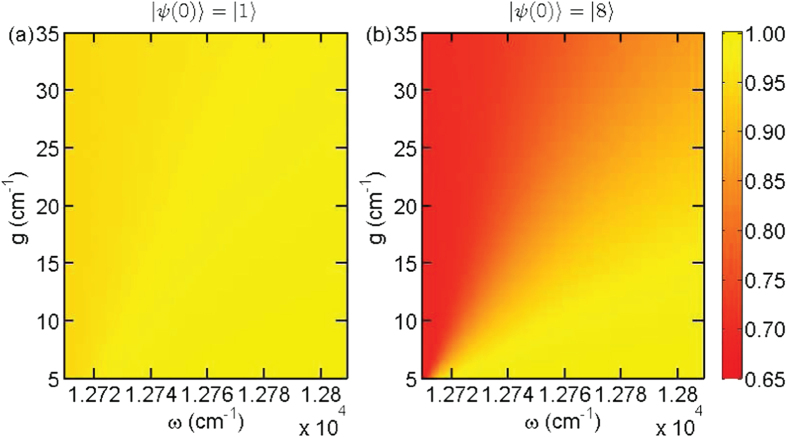
Quantum yield Φ vs the driving frequency *ω* and Rabi frequency *g* for the pulse duration *T* = 170 fs and the beginning time *t*_1_ = 50 fs: (**a**) initial state |*ψ*(0)〉 = |1〉 with fluorescence visibility *V* = 0.03; (**b**) |*ψ*(0)〉 = |8〉 with *V* = 0.18.

As shown in Fig. 2, we plot the quantum yield Φ vs the pulse width *T* and Rabi frequency *g* for the driving frequency *ω* = 12709 cm^−1^ and the beginning time *t*_1_ = 50 fs. In Fig. 2(a), for the whole parameter range, the quantum yields are very close to unity, since the applied laser field is largely detuned from the state 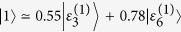. In this case, the population on lower exciton states cannot be transferred to the ground state by means of the laser. In contrast, as illustrated in Fig. 2(b), the detected quantum yields vary significantly for different Rabi frequencies *g* and pulse width *T*. Especially, for a given Rabi frequency, the quantum yield decreases along with the increase of pulse width. That is because more population on the highest exciton state will be resonantly transferred to the ground state through the laser-induced transition as the pulse lasts for a longer duration. Obviously, there is a larger visibility for the case with the initial state  
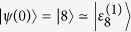
 in comparison to that with 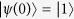, i.e., *V* = 0.16 vs *V* = 0.02, due to the close-resonance condition. This remarkable difference confirms our conjecture that the difference in initial states can be detected by the single-molecule pump-dump experiment. A similar result, i.e., *V* = 0.18 vs *V* = 0.03, is also observed for the quantum yield Φ vs the driving frequency *ω* and the Rabi frequency *g* for the pulse duration *T* = 170 fs and the beginning time *t*_1_ = 50 fs in Fig. 3. We also notice that the dependence of Φ on *ω* and *g* becomes more complicated, shown in Fig. 3(b). When we increase the drive frequency, the quantum yield of fluorescence also raises as the laser field is tuned far away from resonance with the highest exciton state. In this situation, no population on the exciton states can be effectively transferred to the ground state. Besides, for a realistic molecule, there would be fluctuations in the site energies, or the laser field is not exactly perpendicular to the selected transition dipoles, i.e., *g*_60_, *g*_70_ ≠ 0. Even in this case, according to our numerical simulation, the expected visibility for the initial state |8〉 is still significantly larger than that for |1〉. As a practical criterion, we set the median *V*_*m*_ ≃ 0.1 to judge whether the EET path is through |8〉 or not. For a visibility larger than *V*_*m*_, the energy is transferred through |8〉, otherwise it is through |1〉 only.

#### Dissipative two-level system approximation

In order to reveal the underlying physical mechanism, we approximately describe the above experiment by a dissipative two-level system in the closely-resonant case. To be specific, the laser is tuned in close resonance with the transition between the highest-exciton state and the ground state, i.e., 
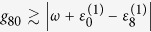
. In this case, the system is governed by two sets of differential equations. For the duration of free evolution, that is


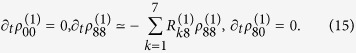


Straightforwardly, at the end of the free evolution, we have


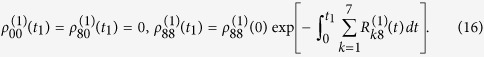


In this situation, the population of the ground state remains unchanged because there is no transition to the ground state induced by either the laser pulse or the system-bath couplings. And the loss of population in the highest exciton state results from its dissipation to the lower exciton states.

On the other hand, for the duration with a laser pulse applied, the equation of motion for the two-level system reads


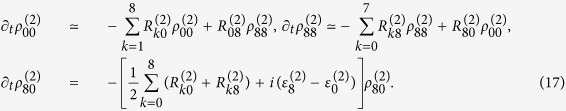


On account of the unitary transformation from the eigen bases in the static frame 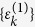 to the eigen bases in the rotating frame 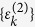, i.e., 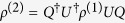, with


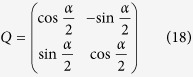


in the bases 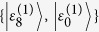, the initial condition is


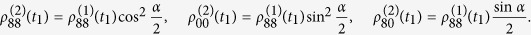


Here, the mixing angle is defined as 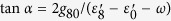. At the end of the pulse duration, the coherence between 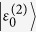 and 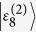 is given by


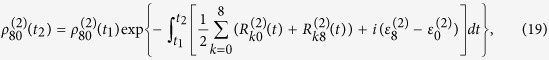


and 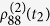 and 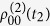 are obtained by solving the first two equations of Eq. (17).

**Figure 4:**
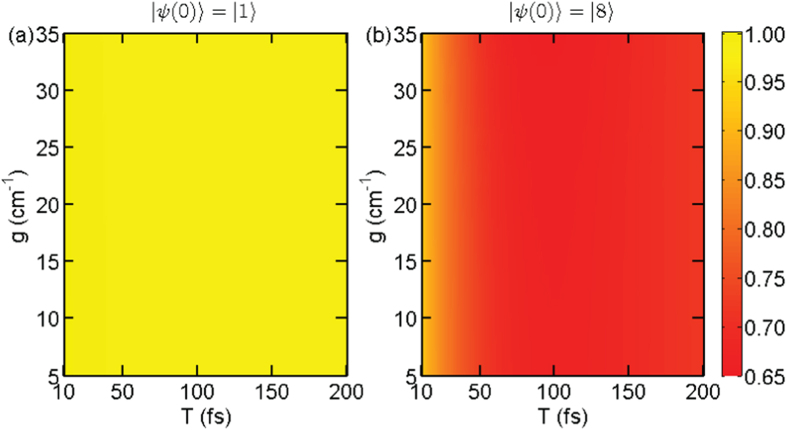
Quantum yield Φ vs *T* and *g* for the dissipative two-level system approximation: (**a**) initial state |*ψ*(0)〉 = |1〉 with *V* = 0.006; (**b**) |*ψ*(0)〉 = |8〉 with *V* = 0.16. All the parameters are the same as those used in Fig. 2.

Based on the dissipative two-level approximation, the numerical evaluation of quantum yield Φ vs Rabi frequency *g* and pulse width *T* is given in Fig. 4. At the first glance, the two sub-figures are similar to the counterparts in Fig. 2. As shown in Fig. 4(b), when we gradually increase the width from zero, the quantum yield quickly drops to nearly one half of the original value. Moveover, as the longer the dump pulse lasts, the more population on 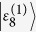 will dumped to the ground state, leading to lower fluorescence yield. However, because the large-detuning condition sets a upper bound for the Rabi frequency, this effectively prevents complete population transfer to the ground state. Moreover, if we further raise the Rabi frequency, the discrepancy between Figs 2(b) and 4(b) will become significant, which is not shown here, since the two-level approximation breaks down in this situation and more exciton states will be probed by the laser. On the other hand, if we extend the pulse duration further, the quantum yield will rise again due to Rabi oscillation. However, dissipation to lower exciton states will eventually erase the Rabi oscillation. This discovery is consistent with our conjecture that the FMO complex prepared initially at |8〉 experiences Rabi oscillation between the highest exciton state and the ground state when there is a laser pulse applied on the system.

## Discussion

As demonstrated in ref. 56, by reducing the number of chlorophylls, the photosynthesis can be optimized in design of artificial light-harvesting. When a chlorophyll is not within the EET pathway, it is redundant to the light-harvesting device. Here, we propose a single-molecule pump-dump experiment scheme for detecting the EET pathway in BChl complexes. By coherently dumping the population on the exciton-states in the EET pathway to the ground state, the energy transfer path in FMO can be determined by detecting fluorescence emission. For a smaller fluorescence visibility *V*, it corresponds to the EET path through site 1 only, as the energy flow through this path has not been probed by the laser due to the large-detuning condition. On the contrary, for a larger *V*, the EET passes through the site 8, because the amount of fluorescence can be tuned by adjusting the frequency and width of the pulse, and the Rabi frequency induced by the laser.

In order to simulate the quantum dynamics in the single-molecule pump-dump experiments, we utilize the newly-developed CMRT-NMQJ approach^9^,^40^. The CMRT describes the quantum dynamics of EET in photosynthetic complexes over a broad parameter regime^41^,^42^ and it is generalized to simulate the energy transfer in the presence of laser fields^40^. Furthermore, the master equation of CMRT recast in Lindblad form can be efficiency solved by the NMQJ method^44^,^45^. Since the CMRT can simulate the absorption spectrum, it can self-consistently obtain the parameters for further simulation^40^. In a recent paper^57^, due to quantum vibrational effects the transfer rate is smaller than that obtained from modified Redfield theory, which may imply that it will take more time for the energy transfer from BChl 8. In this sense, the difference between the visibilities in the cases with or without BChl 8 would probably become more notable. On the other hand, in ref. 41, the population dynamics for FMO are compared by the CMRT and HEOM. Clearly, the coherent dynamics simulated by the HEOM can be well reproduced by the CMRT. Particularly, the population transfer time therein is consistent with that obtained by the HEOM. However, the observable discrepancy lies in the steady-state population. Together with a recently-developed improved variational master equation theory^58^, this problem could be well fixed.

We further remark that the laser-induced Rabi frequency *g*_80_ between the highest exciton state 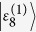 and the ground state  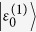 should be sufficiently large when it is compared to their effective level spacing  
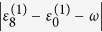
 for a sufficient amount of population to be transferred to the ground state. Meanwhile, the laser-induced Rabi frequencies *g*_*k*0_ between other exciton states  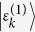 (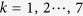) and the ground state 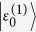 should be sufficiently small when compared to their effective level spacings  
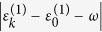
 in order to not transfer the population on the other exciton states to the ground state. In this case, the FMO complex under the quantum control of laser fields experiences Rabi oscillation and relaxation due to the couplings to the bath. In other words, the complex can be well described by a dissipative two-level system under the influence of laser pulses. On the other hand, our scheme is based on the sample where an FMO complex and a baseplate and outer antenna get together. Since it might not be easy to fabricate such a compound complex, an alternative way is to prepare the single-excitation states in the site basis, i.e., |1〉 and |8〉. By using a combination of laser pulses with different physical parameters, e.g., frequency, width, and amplitude, we can effectively prepare the FMO complex in such states with a specific site excited. However, since it is beyond the scope of the current paper, the scheme for state preparation will be presented in a forthcoming paper.

## Methods

The *n*th electric dipole 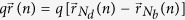 points along the axis connecting the *N*_*b*_ and *N*_*d*_ atoms of *n*th BChl molecule^15^, where


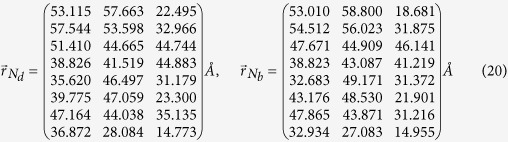


can be obtained from ref. 59. As a result, the transition dipole between the ground state and *k*th exciton state reads 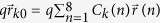.

According to ref. 15, the Hamiltonian for eight-BChls FMO in the single-excitation subspace is


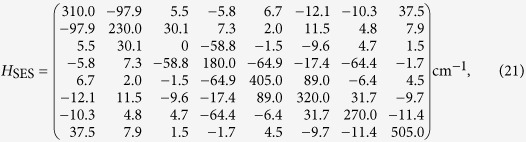


where the energy of ground state is chosen as *E*_0_ = −12195 cm^−1^. On account of the manifold of the ground state, the total Hamiltonian reads 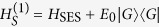, which governs the quantum dynamics of EET in the absence of laser fields.

## Additional Information

**How to cite this article**: Tao, M.-J. *et al*. Proposal for probing energy transfer pathway by single-molecule pump-dump experiment. *Sci. Rep.* **6**, 27535; doi: 10.1038/srep27535 (2016).
